# Causal relationship between type 2 diabetes mellitus and aortic dissection: insights from two-sample Mendelian randomization and mediation analysis

**DOI:** 10.3389/fendo.2024.1405517

**Published:** 2024-05-13

**Authors:** Weizong Zhang, Jindong Sun, Huamin Yu, Minjuan Shi, Haiqiang Hu, Hong Yuan

**Affiliations:** Department of Cardiovascular, First People’s Hospital of LinPing District, Hangzhou, China

**Keywords:** type 2 diabetes mellitus, aortic dissection, causal relationship, mediation analysis, Mendelian randomization, FinnGen

## Abstract

**Objective:**

Some evidence suggests a reduced prevalence of type 2 diabetes mellitus (T2DM) in patients with aortic dissection (AD), a catastrophic cardiovascular illness, compared to general population. However, the conclusions were inconsistent, and the causal relationship between T2DM and AD remains unclear.

**Methods:**

In this study, we aimed to explore the causal relationship between T2DM and AD using bidirectional Mendelian randomization (MR) analysis. Mediation MR analysis was conducted to explore and quantify the possible mediation effects of 1400 metabolites in T2DM and AD.

**Results:**

The results of 26 datasets showed no causal relationship between T2DM and AD (*P*>0.05). Only one dataset (ebi-a-GCST90006934) showed that T2DM was a protective factor for AD (I9-AORTDIS) (OR=0.815, 95%CI: 0.692-0.960, *P*=0.014), and did not show horizontal pleiotropy (*P*=0.808) and heterogeneity (*P*=0.525). Vanillic acid glycine plays a mediator in the causal relationship between T2DM and AD. The mediator effect for vanillic acid glycine levels was -0.023 (95%CI: -0.066-0.021).

**Conclusion:**

From the perspective of MR analysis, there might not be a causal relationship between T2DM and AD, and T2DM might not be a protective factor for AD. If a causal relationship does exist between T2DM and AD, with T2DM serving as a protective factor, vanillic acid glycine may act as a mediator and enhance such a protective effect.

## 1 Introduction

Aortic dissection (AD), a catastrophic cardiovascular illness, occurs when there is an intimal tear that allows the blood to pass through the tear and into the aortic media, splitting the intima in two longitudinally, creating a dissection flap that divides the true lumen from a newly formed false lumen (1). Alarmingly, if AD is not promptly treated, it carries an up to 50% mortality rate within the first 48 hours. Even with treatment, the in-hospital mortality may remain around 10% (2). Consequently, the early identification of AD risk factors is pivotal in improving patient survival rates and prognoses. Research on risk factors for AD has been a hot topic in related disciplines. There are many factors that have been proven to be AD risk factors. Examples: hypertension, males, Marfan syndrome, Turner syndrome, and so on (1).

However, the most specific risk factor is type 2 diabetes mellitus (T2DM). The risk of hypertension in T2DM patients far outweighs that of non-T2DM individuals, and since hypertension is an established risk factor for AD, traditional views categorize T2DM as a potential risk factor for AD. Interestingly though, a 2012 single center case control study in the United States was the first to illustrate a reduced prevalence of T2DM among AD patients, suggesting the potential of T2DM in reducing AD risk, thereby categorizing it as a protective factor (3). This counter intuitive finding was further corroborated through a 2017 meta-analysis including six case control studies, which confirmed, for the first time from an evidence-based medicine standpoint, the negative association between T2DM and AD (4).

T2DM is a complex metabolic disorder that impacts a multitude of metabolic processes and metabolites. A key pathological feature of T2DM is insulin resistance (IR) (5). Study have shown that 20 metabolites such as amino acids, glucose synthesis intermediates, ketone bodies, and fatty acids have a close correlation with IR. At present, the specific mechanism by which T2DM affects AD remains unclear. Few previous studies have examined whether T2DM can affect AD through a certain metabolite. Therefore, we tried to explore the role played by metabolites in T2DM and AD through mediated analysis. Secondly, the genome wide association study (GWAS) data of metabolites are easily available and large, with 1,400 metabolites, making it more likely to find metabolites that act as mediators.

Although these studies analyzed the T2DM and AD association through case control and evidence-based medicine, there remain a few of Mendelian Randomization (MR) analysis studies. Traditional observational studies, impeded by potential confounders and reverse causality, struggle to provide clear causal inferences (6). However, by deploying genetic instrumental variables (IVs) to deduce relationships between exposures and outcomes, MR greatly reduces potential confounder influences upon the validity of association results, enhancing the robustness of the result argument beyond even that of observational studies and randomized controlled trials (7, 8). By adhering to the STROBE-MR (strengthening the reporting of observational studies in epidemiologic trials) statement in this analysis (9), we aim to explore the causal relationship between T2DM and AD, and possible mediators of this effect from an MR perspective, offering fresh insights and evidence into the T2DM-AD causal relationship.

## 2 Materials and methods

### 2.1 Study design

In this study, two-sample MR analysis was used to investigate the potential causal relationship between T2DM (exposure) and AD (outcome). Additionally, reverse MR analysis was performed to determine the causal direction. Mediation MR analysis was conducted to explore and quantify the possible mediation effects of 1400 metabolites in T2DM and AD. The definitions of T2DM and AD were referred to the relevant guidelines (1, 10).

### 2.2 Data sources

Data were sourced from publicly accessible databases (IEU OpenGWAS Project, https://gwas.mrcieu.ac.uk/. FinnGen Release 10, https://r10.finngen.fi/. Diabetes Genetics Replication And Meta-analysis (DIAGRAM) (11), https://diagram-consortium.org/pub.html). Various metabolites GWAS summary statistics were deposited to GWAS Catalog (https://www.ebi.ac.uk/gwas/). Accession numbers for European GWASs: GCST90199621-90201020. These statistics cover a total of 1400 metabolites (12). Because the ethical approvals are described in the original GWAS article, no additional ethical approval was required for the analysis in this study. Data collection is due February 1, 2024.

### 2.3 Selection of IVs

The selecting of IVs was in accordance with the three main hypotheses of MR (8): (1) the IV should be directly associated with the exposure (relevance); (2) the IV should be independent of the confounding factors in the exposure-outcome association (independence); (3) the IV should not have a direct association with the outcome (exclusion). IVs meet the standard of genome wide significance (*P*<5e-6) and clumping for those in linkage disequilibrium (r^2^ = 0.001, distance=10000kb) (13, 14). Instrument strengths were assessed via F-statistics, with high-strength IVs recognized as those with F-statistic>10 and low-strength IVs (F-statistic<10) excluded (15, 16). The 1400 metabolites conformed to genome wide significance (*P*<1e-5) and clumping for those in linkage disequilibrium (r^2^ = 0.001, distance=10000kb).

### 2.4 MR analysis

We mainly used the inverse variance weighted (IVW) method to determine the causal relationship and each potential mediator. Results were shown by odds ratio (OR) and 95% confidence interval (CI). *P<*0.05 was considered to be a potential causal relationship. The “MRPRESSO” package (version 1.0) was employed to identify and remove potential outliers, with reanalysis conducted to ensure hypotheses 2 and 3 were satisfied. Duplicate single nucleotide polymorphism (SNPs) was removed based on rsID, with palindromic SNPs also excluded. A two-sided *P*<0.05 was considered statistically significant. All statistical analysis were performed using the “TwoSampleMR” package (version 0.5.9) in R Software (version 4.3.2).

### 2.5 Mediation effects analysis

The exposure effect on the mediator is defined as beta1, while the mediator effect on the outcome is defined as beta2. Mediator effect (ME) is calculated as ME=beta1*beta2. Total exposure effect on the outcome (TE) and direct exposure effect (DE) are defined as TE=beta_all and DE=beta_all-beta1*beta2 respectively. ME results were shown by 95% CI. Meanwhile, 95% CI were calculated with the delta method (17).

### 2.6 Heterogeneity and pleiotropic analysis

MR-Egger test was employed to test for heterogeneity, and *P<*0.05 indicated the presence of heterogeneity. The Egger intercept method was used to detect the pleiotropic effects of IVs, and the intercept term *P<*0.05 indicated the existence of pleiotropic effects.

## 3 Results

### 3.1 Data collection

A total of 27 T2DM GWAS datasets (sample size=7944837) were collected, except for 2 datasets where case and control were not reported, the remaining 25 datasets contained a total of (case=1071828, control=5337870) (18–33). GWAS meta-analysis summary data (All_Metal_LDSC-CORR_Neff.v2) from DIAGRAM database were included (case=428452, control=2107149) (10). Two GWAS datasets from AD were also included (sample size=589955, case=1437, control=588518) (34). For details, see **Table 1**.

**Table 1:** Baseline characteristics of T2DM and AD datasets.

No.	GWAS ID	Year	Trait	Sources	Population	Sample size (n)	Case (n)	Control (n)
1	ieu-a-24	2012	T2DM	openGWAS	Mixed	149821	34840	114981
2	ebi-a-GCST005047	2012	T2DM	openGWAS	European	69033	6377	5794
3	ieu-a-26	2012	T2DM	openGWAS	European	69033	12171	56862
4	ieu-a-976	2012	T2DM	openGWAS	Mixed	64171	10247	53924
5	ieu-a-23	2014	T2DM	openGWAS	Mixed	110452	26488	83964
6	ieu-a-25	2015	T2DM	openGWAS	Mixed	84780	27206	57574
7	ieu-a-1090	2016	T2DM	openGWAS	European	120286	4040	116246
8	ebi-a-GCST006867	2018	T2DM	openGWAS	European	655666	61714	1178
9	ebi-a-GCST90029024	2018	T2DM	openGWAS	European	468298	—	—
10	ebi-a-GCST007515	2018	T2DM	openGWAS	European	298957	48286	250671
11	ebi-a-GCST007517	2018	T2DM	openGWAS	European	298957	48286	250671
12	ebi-a-GCST005413	2018	T2DM	openGWAS	European	70127	12931	57196
13	ebi-a-GCST005898	2018	T2DM	openGWAS	European	20979	5277	15702
14	bbj-a-153	2019	T2DM	openGWAS	East Asian	210865	40250	170615
15	bbj-a-77	2019	T2DM	openGWAS	East Asian	191764	36614	155150
16	ebi-a-GCST008048	2019	T2DM	openGWAS	Hispanic/Latin American	20480	5971	4135
17	ebi-a-GCST010118	2020	T2DM	openGWAS	East Asian	433540	77418	356122
18	ebi-a-GCST90006934	2020	T2DM	openGWAS	European	22326	9978	12348
19	ebi-a-GCST90018926	2021	T2DM	openGWAS	European	490089	38841	451248
20	ebi-a-GCST90038634	2021	T2DM	openGWAS	European	484598	3260	481338
21	ebi-a-GCST90013892	2021	T2DM	openGWAS	European	406831	—	—
22	finn-b-E4_DM2	2021	T2DM	openGWAS	European	215654	32469	183185
23	finn-b-E4_DM2_STRICT	2021	T2DM	openGWAS	European	212351	29166	183185
24	ebi-a-GCST90018706	2021	T2DM	openGWAS	East Asian	177415	45383	132032
25	ebi-a-GCST90026417	2021	T2DM	openGWAS	European	12230	9486	2744
26	ebi-a-GCST90093109	2022	T2DM	openGWAS	South Asian	50533	16677	33856
27	All_Metal_LDSC-CORR_Neff.v2	2024	T2DM	DIAGRAM	Mixed	2535601	428452	2107149
28	finn-b-I9-AORTDIS	2021	AD	openGWAS	European	382944	967	381977
29	I9-AORTDIS	2023	AD	FinnGen	European	207011	470	206541

T2DM, type 2 diabetes mellitus; AD, aortic dissection; GWAS, genome-wide association studies; DIAGRAM, Diabetes Genetics Replication and Meta-analysis.

### 3.2 MR analysis

**Figure 1:**
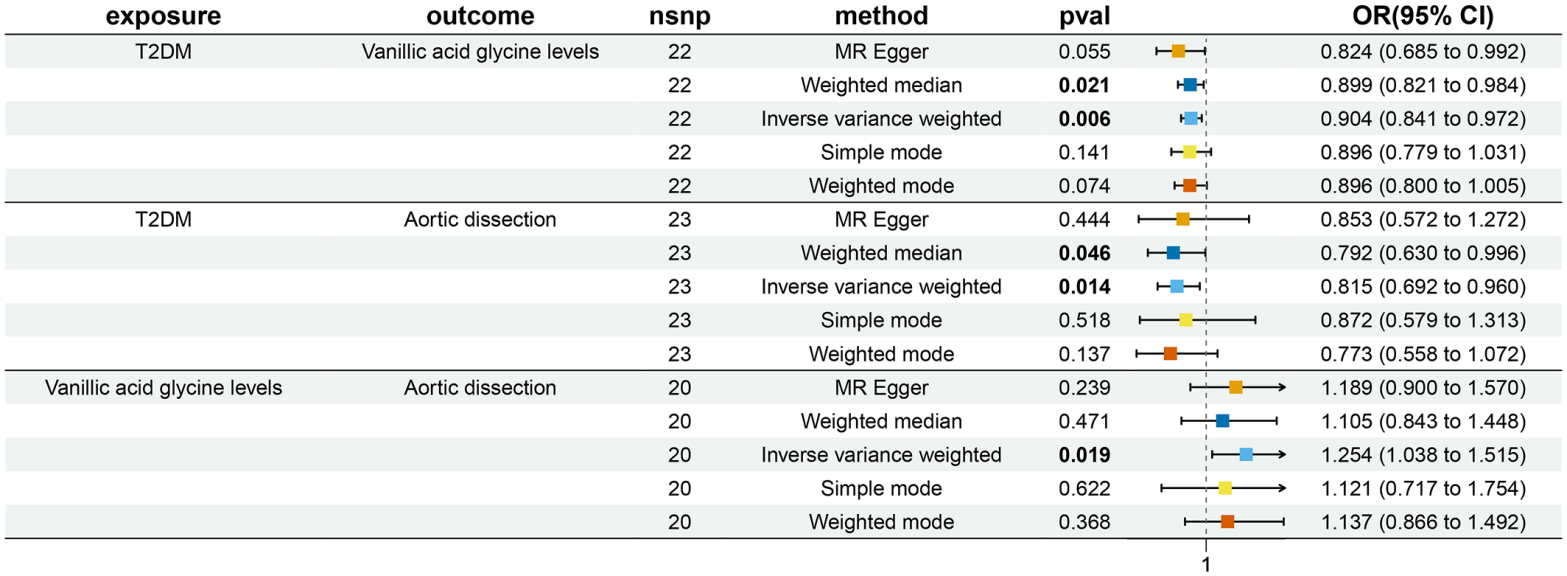
Bidirectional and Mediation MR analysis of T2DM (ebi-a-GCST90006934), vanillic acid glycine levels (ebi-a-GCST90200253) and AD (I9-AORTDIS).

By MR analysis of T2DM and AD, the results of 26 datasets showed no causal relationship between T2DM and AD (*P*>0.05). For details, see **Supplementary Table 1**. Only one diabetes dataset (ebi-a-GCST90006934) showed that T2DM was a protective factor for AD (I9-AORTDIS) (OR=0.815, 95%CI: 0.692-0.960, *P*=0.014), and the results did not show horizontal pleiotropy (*P*=0.808) and heterogeneity (*P*=0.525). For details, see **Table 2**. The eligible IVs are shown in **Supplementary Table 2**, and the correlation analysis plots are shown in **Supplementary Figure 1**. The reverse MR analysis suggested that there was no causal relationship between AD (I9-AORTDIS) and T2DM (ebi-a-GCST90006934) (OR=1.026, 95%CI: 0.976-1.079, *P*=0.320), and the result was free of horizontal pleiotropy (*P*=0.172) and heterogeneity (*P*=0.444). For details, see **Table 2**. Suggesting that the causal direction was T2DM to AD. The forest plots are shown in **Figure 1**, the eligible IVs are shown in **Supplementary Table 3**, and the correlation analysis plots are shown in the **Supplementary Figure 2**.

**Table 2:** MR analysis of ebi-a-GCST90006934, I9-AORTDIS, finn-b-I9-AORTDIS and ebi-a-GCST90200253.

Exposure	Outcome	Method	SNP (n)	beta	OR	*P*	95%CI		Pleiotropy Test	Heterogeneity Test
									*P-ple*	*P-het*
ebi-a-GCST90006934	I9-AORTDIS	MR Egger	23	-0.159	0.853	0.444	0.572	-1.272	0.808	0.525
		Weighted median	23	-0.233	0.792	0.046	0.630	-0.996		
		IVW	23	-0.205^c^	0.815	0.014	0.692	-0.960		
		Simple mode	23	-0.137	0.872	0.518	0.579	-1.313		
		Weighted mode	23	-0.257	0.773	0.137	0.558	-1.072		
I9-AORTDIS	ebi-a-GCST90006934	MR Egger	15	0.098	1.103	0.103	0.988	-1.231	0.172	0.444
		Weighted median	15	0.019	1.019	0.605	0.948	-1.095		
		IVW	15	0.026	1.026	0.320	0.976	-1.079		
		Simple mode	15	0.016	1.016	0.831	0.879	-1.175		
		Weighted mode	15	0.107	1.113	0.160	0.966	-1.283		
ebi-a-GCST90006934	finn-b-I9-AORTDIS	MR Egger	23	-0.003	0.997	0.992	0.536	-1.853		
		Weighted median	23	-0.365	0.694	0.040	0.490	-0.983		
		IVW	23	-0.296	0.744	0.022	0.578	-0.958	0.322	0.281
		Simple mode	23	-0.623	0.537	0.109	0.259	-1.113		
		Weighted mode	23	-0.279	0.757	0.321	0.442	-1.296		
ebi-a-GCST90006934	ebi-a-GCST90200253	MR Egger	22	-0.193	0.824	0.055	0.685	-0.992	0.302	0.096
		Weighted median	22	-0.106	0.899	0.021	0.821	-0.984		
		IVW	22	-0.101^a^	0.904	0.006	0.841	-0.972		
		Simple mode	22	-0.109	0.896	0.141	0.779	-1.031		
		Weighted mode	22	-0.109	0.896	0.074	0.800	-1.005		
ebi-a-GCST90200253	I9-AORTDIS	MR Egger	20	0.173	1.189	0.239	0.900	-1.570	0.607	0.285
		Weighted median	20	0.100	1.105	0.471	0.843	-1.448		
		IVW	20	0.226^b^	1.254	0.019	1.038	-1.515		
		Simple mode	20	0.114	1.121	0.622	0.717	-1.754		
		Weighted mode	20	0.128	1.137	0.368	0.866	-1.492		

MR, Mendelian randomization; IVW, Inverse variance weighted; SNP, single nucleotide polymorphism; OR, odds ratio; CI, confidence interval; Pleiotropy Test use Egger intercept method; Heterogeneity Test use MR-Egger test; a: beta1; b: beta2; c: beta_all.

### 3.3 MR validation

By using AD (finn-b-I9-AORTDIS) for validation, the results of MR analysis of the 26 T2DM datasets showed that T2DM and AD were not causally related (*P*>0.05). For details, see **Supplementary Table 1**. Either only ebi-a-GCST90006934 showed that T2DM was a protective factor for AD (finn-b-I9-AORTDIS) (OR=0.744, 95%CI: 0.578-0.958, *P*=0.022), and the results were not subject to horizontal pleiotropy (*P*=0.322) and heterogeneity (*P*=0.281). For details, see **Table 2**. The eligible IVs are shown in **Supplementary Table 2**, and the correlation analysis plots are shown in **Supplementary Figure 3**. The reverse MR analysis of AD (finn-b-I9-AORTDIS) to T2DM (ebi-a-GCST90006934) was not performed because AD (finn-b-I9-AORTDIS) was unable to screen out the strong IVs. The above MR analysis indicates that there may not be a causal relationship between T2DM and AD. The results of MR analysis of only one dataset with T2DM supported that T2DM was a protective factor for AD. The forest plot is shown in **Figure 1**.

### 3.4 Selection of mediator from 1400 metabolites

The ebi-a-GCST90200253 dataset was found to be causally associated with T2DM (ebi-a-GCST90006934) and AD (I9-AORTDIS) by screening 1400 metabolites, respectively, and the results were not horizontally pleiotropic (*P*=0.322) and heterogeneous (*P*=0.281), as shown in **Table 2**. The ebi-a-GCST90200253 matching metabolites was vanillic acid glycine levels. This suggests that vanillic acid glycine plays a mediating role in the causal relationship between T2DM and AD. The forest plot is shown in **Figure 1**.

### 3.5 Mediation effects of mediator

From the calculations, we derived beta1=-0.101 (OR=0.904, 95%CI: 0.841-0.972, *P*=0.006), beta2 = 0.226 (OR=1.254, 95%CI: 1.038-1.515, *P*=0.019), beta_all=-0.205 (OR=0.815, 95%CI: 0.692-0.960, *P*=0.014), see **Table 2**, which gives TE=-0.025, which suggests that AD decreases when T2DM increases. The DE =-0.182, which suggests that increasing T2DM leads to a decrease in AD even without the mediating variable of vanillic acid glycine levels. The ME for vanillic acid glycine levels was -0.023 (95%CI: -0.066-0.021), which suggests that an increase in T2DM leads to a decrease in AD through vanillic acid glycine leads to a decrease in AD.

## 4 Discussion

Our research indicates the possible lack of a causal relationship between T2DM and AD, suggesting that T2DM may not play a protective role in AD. Supposing a causal relationship does exist between T2DM and AD, our findings propose that T2DM could serve as a protective factor, with vanillic acid glycine acting as a mediator to enhance this protective impact.

Numerous observational studies and meta-analyses have been undertaken to explore the association between T2DM and AD. The impact of T2DM on the morbidity of AD was first highlighted by a single center case control study in the United States published by Journal of the American Heart Association (JAHA) in 2012 (3). The study indicated a lower prevalence of T2DM among AD patients, implying that T2DM may mitigate the risk of AD, thus potentially declaring T2DM as a protective factor against AD. Complementing these findings, a meta-analysis collecting for six relevant case control studies was published in Angiology in 2017, confirming a negative correlation between T2DM and AD (4). A subsequent large cohort study by JAHA in 2018 (35), possibly the most extensive of its kind, also evidenced a negative correlation between T2DM and AD, drawing from a massive sample size comprising 448,319 T2DM patients and 2,251,015 control participants spanning from 1998 to 2015. The revelation came with the data showing that T2DM patients had a 47% reduced relative risk of AD when compared to the control group (hazard ratio=0.53, 95% CI: 0.42-0.65, *P*<0.0001). Nonetheless, these studies fail to provide a reason for the increasing prevalence of both T2DM and AD in recent years (36, 37). Logically, if T2DM does serve as a protective factor, the incidence of AD should have either dropped or remained static as T2DM cases rose. This question was also raised by the famous scholar, Nienaber, in 2021 (38). A recent meta-analysis collecting for 14 relevant studies suggests yet again that T2DM could protect against AD, although the study’s high heterogeneity (*I*^2^ = 86.5%) and unprobed source of heterogeneity could lead to a biased conclusion (39). Furthermore, a subgroup analysis of population groups exhibited varied results: among non-Chinese populations, T2DM could serve as a protective factor (OR=0.45, 95%CI: 0.27-0.74, *I*^2^ = 58.4%), whereas no such association, coupled with high heterogeneity, was observed among Chinese populations (OR=0.59, 95%CI: 0.26-1.33, *I*^2^ = 93.5%). These observations indicate potential population-based variations on the influence of T2DM on the incidence of AD, particularly pointing to the unlikely protective factor of T2DM against AD in the Chinese population.

Regarding the prognostic influence of T2DM on AD patients, Avdic et al. (35) observed the mortality rate among T2DM patients remained unchanged within 2 years post-hospitalization for AD. Similarly, He et al. (40) categorized AD patients from a Chinese cohort based on T2DM, and found no statistical significance in the in-hospital mortality rates between these two groups. In alignment with this finding, Chen et al. (41) also reported no significant difference in the 30-day mortality rate between AD patients with or without T2DM. Even over a median follow-up period of 21.3 months, mortality rates did not significantly differ between the two groups, as confirmed by a multifactorial COX regression analysis. However, there were conflicting results from Jiménez-Trujillo et al. (42) where a Spanish cohort showed significantly lower in-hospital mortality in AD patients with T2DM. Liu et al. (43) reviewed aortic endovascular repair (TEVAR) outcomes in AD patients with T2DM and found a significant reduction in postoperative mortality and clinical complications over a 3-year follow-up period. Based on these varying findings, a consistent conclusion regarding the influence of T2DM on the prognosis of AD patients remains elusive.

The observational studies mentioned above are inevitably affected by potential confounders and reverse causality. This is where MR analysis can take full advantage of its strengths and provide important clues. To the best of our knowledge, our study is the first to explore the causal relationship between T2DM and AD through MR analysis. In this study, we avoided the selection bias brought about by the artificial selection of appropriate data sets by using the scheme of analyzing 27 T2DM data sets one by one. We also used 2 AD datasets for mutual validation, which resulted in higher reliability and stability of the conclusions. We also excluded the effect of reverse causality by reverse MR analysis. In our study, only one dataset of T2DM was analyzed to support the existence of a causal relationship between T2DM and AD, and the rest of the datasets did not support this conclusion. This suggests that there may not be a causal relationship between T2DM and AD and that T2DM may not be a protective factor for AD. There are several possible explanations for this. First, it may be related to the small effect of SNPs on T2DM (i.e., they explain only a small fraction of the variance). Second, there may be differences between the “ebi-a-GCST90006934” dataset and other datasets (For example: sample size, population, age, gender composition, etc.). Third, combining the results of previous observational studies and meta-analyses, it can be inferred that the effect of T2DM on AD patients may be achieved more through the acquired environment, behaviors after the disease, and taking medications. The dataset used in this study has a wide time span and covers a wide range of populations, making the conclusions extrapolatable. For the ebi-a-GCST90006934 dataset, we used a mediation analysis to explore the role of 1400 metabolites in the causal relationship between T2DM and AD. We found that if there is a causal relationship between T2DM and AD, T2DM is a protective factor for AD, and that vanillic acid glycine mediates and enhances this protective effect. Although there are fewer studies of vanillic acid glycine in T2DM and AD and the exact mechanism of action is unclear, this may be more exploratory.

Although the relationship between vanillic acid glycine, T2DM, and AD is not clear yet, current studies show that vanillic acid and glycine are protective factors for T2DM (44, 45). Depletion of glycine may expose pancreatic cells to oxidative stress, resulting in the loss of the pancreas’s compensatory mechanism for hyperglycemia (44). The relationship between T2DM and AD is still controversial, and the mechanism is not clear. However, possible explanations include: 1. Diabetes can promote the synthesis and reduce the degradation of the extracellular matrix by reducing the expression of matrix metalloproteinases and increasing advanced glycation end products in the extracellular matrix, while advanced glycation end products help thicken the aortic wall (46). 2. Thiazolidinediones and metformin, and other diabetes drugs can achieve a protective effect against AD by reducing the expression of matrix metalloproteinases in the aortic wall. Therefore, we can hypothesize that due to the reduction of protective factors such as vanillic acid and glycine, the incidence of T2DM increases, while T2DM reduces the incidence of AD through related mechanisms. The relationship between vanillic acid glycine, T2DM, and AD is complex and needs more studies to confirm. In therapeutic decisions and public health interventions, attention can be paid to the changes in vanillic acid and glycine. At the same time, because the relationship between T2DM and AD is still under debate, it can’t be rashly assumed that T2DM is a protective factor for AD, and T2DM still needs to be actively treated.

Limitations of this study: 1. Although our study followed certain criteria to screen the IV, there may be differences in the results obtained if these criteria are changed, which would require a larger amount of data and more analytical methods to be validated. 2. This study screened for metabolites for which there are fewer relevant studies. 3. The number of patients varies widely in AD and T2DM, which may lead to a decrease in statistical efficacy.

The above discussion shows that the relationship between T2DM and AD may be influenced by genetic variation, acquired environment, and different populations at the same time. In addition, both T2DM and AD are diseases with complex pathophysiologic mechanisms, and studies with more perspectives and larger sample sizes are needed to confirm the causal relationship between them.

## 5 Conclusion

From the perspective of MR analysis, there might not be a causal relationship between T2DM and AD, and T2DM might not be a protective factor for AD. If a causal relationship does exist between T2DM and AD, with T2DM serving as a protective factor, vanillic acid glycine may act as a mediator and enhance such a protective effect.

## Data availability statement

The original contributions presented in the study are included in the article/**Supplementary Material**. Further inquiries can be directed to the corresponding author.

## Ethics statement

Ethical approval was not required for the study involving humans in accordance with the local legislation and institutional requirements. Written informed consent to participate in this study was not required from the participants or the participants’ legal guardians/next of kin in accordance with the national legislation and the institutional requirements.

## Author contributions

WZ: Writing – original draft. JS: Writing – review & editing. HMY: Writing – review & editing. MS: Writing – review & editing. HH: Writing – review & editing. HY: Writing – review & editing.
