# Supplementary material for: Causal relationship between type 2 diabetes mellitus and aortic dissection: insights from two-sample Mendelian randomization and mediation analysis
Source: Front Endocrinol (Lausanne). 2024 May 13;15:1405517. doi: 10.3389/fendo.2024.1405517 (PMC11128602; doi:10.3389/fendo.2024.1405517)
Supplement: Supplementary file 1 [file DataSheet_1.docx]

Supplementary Material

# Supplementary Tables

| **Supplementary Table 1.** Bidirectional MR study of association between T2DM and AD | | | | | | | |
| --- | --- | --- | --- | --- | --- | --- | --- |
| Exposure | Outcome | Method | SNP (n) | OR | *P* | Pleiotropy Test | Heterogeneity Test |
|  |  |  |  |  |  | *P-ple* | *P-het* |
| ieu-a-24 | I9-AORTDIS | — | 0 | — | — | — | — |
| ebi-a-GCST005047 |  | — | 0 | — | — | — | — |
| ieu-a-26 |  | — | 0 | — | — | — | — |
| ieu-a-976 |  | — | 0 | — | — | — | — |
| ieu-a-23 |  | — | 0 | — | — | — | — |
| ieu-a-25 |  | IVW | 28 | 0.854 | 0.072 | 0.341 | 0.381 |
| ieu-a-1090 |  | IVW | 47 | 1.012 | 0.630 | 0.776 | 0.162 |
| ebi-a-GCST006867 |  | IVW | 199 | 0.947 | 0.414 | 0.140 | 0.044 |
| ebi-a-GCST90029024 |  | IVW | 139 | 2.973 | 0.459 | 0.013 | 0.350 |
| ebi-a-GCST007515 |  | IVW | 79 | 1.001 | 0.987 | 0.026 | 0.168 |
| ebi-a-GCST007517 |  | IVW | 66 | 1.027 | 0.828 | 0.037 | 0.029 |
| ebi-a-GCST005413 |  | IVW | 43 | 0.902 | 0.128 | 0.276 | 0.385 |
| ebi-a-GCST005898 |  | — | 0 | — | — | — | — |
| bbj-a-153 |  | — | 0 | — | — | — | — |
| bbj-a-77 |  | — | 0 | — | — | — | — |
| ebi-a-GCST008048 |  | IVW | 38 | 0.926 | 0.366 | 0.994 | 0.056 |
| ebi-a-GCST010118 |  | IVW | 218 | 0.933 | 0.241 | 0.093 | 0.712 |
| ebi-a-GCST90006934 |  | IVW | 23 | 0.815 | 0.014 | 0.808 | 0.525 |
| ebi-a-GCST90018926 |  | IVW | 284 | 0.947 | 0.353 | 0.120 | 0.053 |
| ebi-a-GCST90038634 |  | IVW | 25 | 0.001 | 0.582 | 0.355 | 0.128 |
| ebi-a-GCST90013892 |  | — | 0 | — | — | — | — |
| finn-b-E4_DM2 |  | — | 0 | — | — | — | — |
| finn-b-E4_DM2_STRICT |  | — | 0 | — | — | — | — |
| ebi-a-GCST90018706 |  | IVW | 148 | 0.997 | 0.959 | 0.587 | 0.098 |
| ebi-a-GCST90026417 |  | IVW | 12 | 0.929 | 0.414 | 0.480 | 0.207 |
| ebi-a-GCST90093109 |  | IVW | 56 | 0.943 | 0.470 | 0.116 | 0.139 |
| All_Metal_LDSC-CORR_Neff.v2 |  | IVW | 642 | 0.941 | 0.363 | 0.021 | 0.028 |
| I9-AORTDIS | ieu-a-24 | — | 0 | — | — | — | — |
|  | ebi-a-GCST005047 | IVW | 2 | 0.999 | 0.992 | NA | 0.267 |
|  | ieu-a-26 | WR | 1 | 0.965 | 0.569 | NA | NA |
|  | ieu-a-976 | WR | 1 | 0.888 | 0.148 | NA | NA |
|  | ieu-a-23 | IVW | 2 | 0.999 | 0.981 | NA | 0.288 |
|  | ieu-a-25 | — | 0 | — | — | — | — |
|  | ieu-a-1090 | IVW | 12 | 0.986 | 0.831 | 0.684 | 0.121 |
|  | ebi-a-GCST006867 | IVW | 8 | 0.978 | 0.138 | 0.187 | 0.230 |
|  | ebi-a-GCST90029024 | IVW | 15 | 1.000 | 0.630 | 0.236 | 0.422 |
|  | ebi-a-GCST007515 | WR | 1 | 0.996 | 0.886 | NA | NA |
|  | ebi-a-GCST007517 | WR | 1 | 0.972 | 0.427 | NA | NA |
|  | ebi-a-GCST005413 | IVW | 14 | 0.990 | 0.659 | 0.671 | 0.218 |
|  | ebi-a-GCST005898 | — | 0 | — | — | — | — |
|  | bbj-a-153 | IVW | 10 | 1.010 | 0.443 | 0.312 | 0.088 |
|  | bbj-a-77 | IVW | 9 | 1.006 | 0.680 | 0.449 | 0.060 |
|  | ebi-a-GCST008048 | IVW | 16 | 0.974 | 0.129 | 0.069 | 0.870 |
|  | ebi-a-GCST010118 | IVW | 10 | 1.001 | 0.356 | 0.654 | 0.123 |
|  | ebi-a-GCST90006934 | IVW | 15 | 1.026 | 0.320 | 0.172 | 0.444 |
|  | ebi-a-GCST90018926 | IVW | 10 | 1.011 | 0.359 | 0.418 | 0.230 |
|  | ebi-a-GCST90038634 | IVW | 14 | 1.000 | 0.225 | 0.981 | 0.077 |
|  | ebi-a-GCST90013892 | IVW | 16 | 0.997 | 0.840 | 0.009 | 0.458 |
|  | finn-b-E4_DM2 | IVW | 15 | 0.977 | 0.184 | 0.065 | 0.125 |
|  | finn-b-E4_DM2_STRICT | IVW | 15 | 0.980 | 0.197 | 0.071 | 0.337 |
|  | ebi-a-GCST90018706 | IVW | 10 | 1.004 | 0.831 | 0.215 | 0.094 |
|  | ebi-a-GCST90026417 | IVW | 8 | 1.044 | 0.473 | 0.347 | 0.203 |
|  | ebi-a-GCST90093109 | IVW | 13 | 0.992 | 0.780 | 0.030 | 0.420 |
|  | All_Metal_LDSC-CORR_Neff.v2 | IVW | 12 | 0.992 | 0.064 | 0.353 | 0.512 |
| ieu-a-24 | finn-b-I9-AORTDIS | — | 0 | — | — | — | — |
| ebi-a-GCST005047 |  | — | 0 | — | — | — | — |
| ieu-a-26 |  | — | 0 | — | — | — | — |
| ieu-a-976 |  | — | 0 | — | — | — | — |
| ieu-a-23 |  | — | 0 | — | — | — | — |
| ieu-a-25 |  | IVW | 28 | 0.800 | 0.067 | 0.883 | 0.503 |
| ieu-a-1090 |  | IVW | 46 | 0.983 | 0.608 | 0.429 | 0.368 |
| ebi-a-GCST006867 |  | IVW | 201 | 0.924 | 0.418 | 0.296 | 0.022 |
| ebi-a-GCST90029024 |  | IVW | 140 | 3.387 | 0.587 | 0.285 | 0.043 |
| ebi-a-GCST007515 |  | IVW | 81 | 0.952 | 0.770 | 0.150 | 0.011 |
| ebi-a-GCST007517 |  | IVW | 68 | 0.940 | 0.741 | 0.327 | 0.001 |
| ebi-a-GCST005413 |  | IVW | 44 | 0.890 | 0.268 | 0.824 | 0.125 |
| ebi-a-GCST005898 |  | — | 0 | — | — | — | — |
| bbj-a-153 |  | — | 0 | — | — | — | — |
| bbj-a-77 |  | — | 0 | — | — | — | — |
| ebi-a-GCST008048 |  | IVW | 37 | 0.999 | 0.993 | 0.817 | 0.059 |
| ebi-a-GCST010118 |  | IVW | 226 | 0.899 | 0.200 | 0.884 | 0.655 |
| ebi-a-GCST90006934 |  | IVW | 23 | 0.744 | 0.022 | 0.322 | 0.281 |
| ebi-a-GCST90018926 |  | IVW | 295 | 1.016 | 0.848 | 0.144 | 0.013 |
| ebi-a-GCST90038634 |  | IVW | 25 | 0.029 | 0.819 | 0.770 | 0.301 |
| ebi-a-GCST90013892 |  | — | 0 | — | — | — | — |
| finn-b-E4_DM2 |  | — | 0 | — | — | — | — |
| finn-b-E4_DM2_STRICT |  | — | 0 | — | — | — | — |
| ebi-a-GCST90018706 |  | IVW | 153 | 1.025 | 0.768 | 0.015 | 0.075 |
| ebi-a-GCST90026417 |  | IVW | 12 | 1.104 | 0.390 | 0.990 | 0.831 |
| ebi-a-GCST90093109 |  | IVW | 56 | 0.941 | 0.639 | 0.216 | 0.007 |
| All_Metal_LDSC-CORR_Neff.v2 |  | IVW | 648 | 0.930 | 0.441 | 0.034 | 0.105 |
| finn-b-I9-AORTDIS | ieu-a-24 | — | 0 | — | — | — | — |
|  | ebi-a-GCST005047 | — | 0 | — | — | — | — |
|  | ieu-a-26 | — | 0 | — | — | — | — |
|  | ieu-a-976 | — | 0 | — | — | — | — |
|  | ieu-a-23 | — | 0 | — | — | — | — |
|  | ieu-a-25 | — | 0 | — | — | — | — |
|  | ieu-a-1090 | — | 0 | — | — | — | — |
|  | ebi-a-GCST006867 | — | 0 | — | — | — | — |
|  | ebi-a-GCST90029024 | — | 0 | — | — | — | — |
|  | ebi-a-GCST007515 | — | 0 | — | — | — | — |
|  | ebi-a-GCST007517 | — | 0 | — | — | — | — |
|  | ebi-a-GCST005413 | — | 0 | — | — | — | — |
|  | ebi-a-GCST005898 | — | 0 | — | — | — | — |
|  | bbj-a-153 | — | 0 | — | — | — | — |
|  | bbj-a-77 | — | 0 | — | — | — | — |
|  | ebi-a-GCST008048 | — | 0 | — | — | — | — |
|  | ebi-a-GCST010118 | — | 0 | — | — | — | — |
|  | ebi-a-GCST90006934 | — | 0 | — | — | — | — |
|  | ebi-a-GCST90018926 | — | 0 | — | — | — | — |
|  | ebi-a-GCST90038634 | — | 0 | — | — | — | — |
|  | ebi-a-GCST90013892 | — | 0 | — | — | — | — |
|  | finn-b-E4_DM2 | — | 0 | — | — | — | — |
|  | finn-b-E4_DM2_STRICT | — | 0 | — | — | — | — |
|  | ebi-a-GCST90018706 | — | 0 | — | — | — | — |
|  | ebi-a-GCST90026417 | — | 0 | — | — | — | — |
|  | ebi-a-GCST90093109 | — | 0 | — | — | — | — |
|  | All_Metal_LDSC-CORR_Neff.v2 | — | 0 | — | — | — | — |
| MR: Mendelian randomization; T2DM: type 2 diabetes mellitus; AD: aortic dissection; SNP: single nucleotide polymorphism; OR: odds ratio; WR: Wald ratio; IVW: inverse-variance weighted; NA: not available; Pleiotropy Test use Egger intercept method; Heterogeneity Test use MR-Egger test. | | | | | | | |

| **Supplementary Table 2.** List of ebi-a-GCST90006934 genetic instrumental variables | | | | | | | | | | | |
| --- | --- | --- | --- | --- | --- | --- | --- | --- | --- | --- | --- |
| id.exposure | SNP | chr | effect_allele | other_allele | eaf | beta | se | pval | samplesize | R^2^ | F |
| ebi-a-GCST90006934 | rs3768321 | 1 | T | G | 0.2068 | 0.1134 | 0.0248 | 4.73E-06 | 22326 | 0.000935632 | 20.90662074 |
| ebi-a-GCST90006934 | rs713311 | 1 | T | C | 0.0708 | -0.1814 | 0.0395 | 4.34E-06 | 22326 | 0.000943755 | 21.08829497 |
| ebi-a-GCST90006934 | rs7546395 | 1 | C | T | 0.4923 | 0.1054 | 0.0223 | 2.39E-06 | 22326 | 0.0009996 | 22.33739835 |
| ebi-a-GCST90006934 | rs952227 | 2 | G | A | 0.6641 | 0.1113 | 0.0211 | 1.31E-07 | 22326 | 0.001244725 | 27.82188246 |
| ebi-a-GCST90006934 | rs73021345 | 3 | A | G | 0.1201 | -0.1492 | 0.0314 | 2.06E-06 | 22326 | 0.001010249 | 22.57560738 |
| ebi-a-GCST90006934 | rs4130874 | 3 | C | T | 0.6644 | -0.0977 | 0.0209 | 3.11E-06 | 22326 | 0.000977824 | 21.8503123 |
| ebi-a-GCST90006934 | rs113617270 | 4 | T | C | 0.0188 | -0.3503 | 0.0755 | 3.46E-06 | 22326 | 0.00096329 | 21.52521336 |
| ebi-a-GCST90006934 | rs552719 | 4 | T | C | 0.1418 | -0.1337 | 0.029 | 3.88E-06 | 22326 | 0.000951136 | 21.25337535 |
| ebi-a-GCST90006934 | rs1549182 | 5 | A | G | 0.5208 | -0.0972 | 0.0201 | 1.30E-06 | 22326 | 0.001046345 | 23.38306885 |
| ebi-a-GCST90006934 | rs149736582 | 5 | T | C | 0.0102 | 0.5537 | 0.1213 | 4.99E-06 | 22326 | 0.00093242 | 20.83476176 |
| ebi-a-GCST90006934 | rs7756992 | 6 | G | A | 0.2851 | 0.1423 | 0.0218 | 6.84E-11 | 22326 | 0.001904837 | 42.60473873 |
| ebi-a-GCST90006934 | rs4300038 | 8 | A | G | 0.2931 | -0.1341 | 0.0221 | 1.24E-09 | 22326 | 0.001646442 | 36.81578811 |
| ebi-a-GCST90006934 | rs146580015 | 8 | T | C | 0.0124 | 0.5475 | 0.1153 | 2.07E-06 | 22326 | 0.001008928 | 22.5460635 |
| ebi-a-GCST90006934 | rs34356122 | 8 | T | C | 0.2047 | 0.1173 | 0.0248 | 2.21E-06 | 22326 | 0.001001029 | 22.36937015 |
| ebi-a-GCST90006934 | rs10811660 | 9 | A | G | 0.18 | -0.1397 | 0.0261 | 9.14E-08 | 22326 | 0.001281575 | 28.64658727 |
| ebi-a-GCST90006934 | rs7903146 | 10 | T | C | 0.3207 | 0.2807 | 0.0216 | 1.53E-38 | 22326 | 0.007507471 | 168.8645225 |
| ebi-a-GCST90006934 | rs1977833 | 10 | A | G | 0.3917 | -0.1318 | 0.0206 | 1.45E-10 | 22326 | 0.001830164 | 40.93148236 |
| ebi-a-GCST90006934 | rs2237895 | 11 | C | A | 0.4257 | 0.0929 | 0.0203 | 4.85E-06 | 22326 | 0.000937176 | 20.94114604 |
| ebi-a-GCST90006934 | rs11603349 | 11 | C | T | 0.1494 | -0.1337 | 0.0279 | 1.70E-06 | 22326 | 0.001027536 | 22.96230607 |
| ebi-a-GCST90006934 | rs143017296 | 14 | T | G | 0.0221 | 0.3349 | 0.0676 | 7.21E-07 | 22326 | 0.001098118 | 24.54132442 |
| ebi-a-GCST90006934 | rs9972653 | 16 | T | G | 0.4235 | 0.1081 | 0.0201 | 7.13E-08 | 22326 | 0.001293856 | 28.92147022 |
| ebi-a-GCST90006934 | rs8056223 | 16 | G | T | 0.0614 | -0.2237 | 0.045 | 6.49E-07 | 22326 | 0.001105645 | 24.70973194 |
| ebi-a-GCST90006934 | rs72999033 | 19 | T | C | 0.0677 | 0.1904 | 0.0394 | 1.33E-06 | 22326 | 0.001044904 | 23.3508416 |
| SNP: single nucleotide polymorphism; chr: chromosome; eaf: effect allele frequency; se: standard error of beta. | | | | | | | | | | | |

| **Supplementary Table 3.** List of I9-AORTDIS genetic instrumental variables | | | | | | | | | | | |
| --- | --- | --- | --- | --- | --- | --- | --- | --- | --- | --- | --- |
| id.exposure | SNP | chr | effect_allele | other_allele | eaf | beta | se | pval | samplesize | R^2^ | F |
| I9-AORTDIS | rs190213906 | 1 | T | C | 0.0528667 | 0.440265 | 0.0901192 | 1.03E-06 | 382944 | 6.23E-05 | 23.86664642 |
| I9-AORTDIS | rs656529 | 1 | G | A | 0.770605 | -0.241825 | 0.0516859 | 2.89E-06 | 382944 | 5.72E-05 | 21.89051398 |
| I9-AORTDIS | rs72794813 | 2 | C | T | 0.0780472 | 0.382315 | 0.0768654 | 6.56E-07 | 382944 | 6.46E-05 | 24.73879969 |
| I9-AORTDIS | rs111385809 | 2 | G | A | 0.0505072 | -0.556656 | 0.121297 | 4.45E-06 | 382944 | 5.50E-05 | 21.06063209 |
| I9-AORTDIS | rs138346871 | 4 | T | G | 0.0049253 | 1.0794 | 0.234981 | 4.36E-06 | 382944 | 5.51E-05 | 21.10070976 |
| I9-AORTDIS | rs72822047 | 5 | G | A | 0.00294019 | 1.31071 | 0.27881 | 2.59E-06 | 382944 | 5.77E-05 | 22.10010132 |
| I9-AORTDIS | rs117776783 | 8 | T | G | 0.00360648 | 1.27046 | 0.260331 | 1.06E-06 | 382944 | 6.22E-05 | 23.81595223 |
| I9-AORTDIS | rs12336187 | 9 | A | G | 0.521788 | 0.221834 | 0.0452904 | 9.68E-07 | 382944 | 6.26E-05 | 23.99062927 |
| I9-AORTDIS | rs79958663 | 10 | T | C | 0.130806 | 0.285541 | 0.0623886 | 4.72E-06 | 382944 | 5.47E-05 | 20.94711436 |
| I9-AORTDIS | rs145146588 | 15 | C | T | 0.018022 | 0.693564 | 0.136348 | 3.64E-07 | 382944 | 6.76E-05 | 25.87457787 |
| I9-AORTDIS | rs36029774 | 15 | T | C | 0.0650941 | 0.545663 | 0.0785183 | 3.67E-12 | 382944 | 1.26E-04 | 48.29531114 |
| I9-AORTDIS | rs939430 | 18 | A | G | 0.656715 | -0.228378 | 0.0466715 | 9.92E-07 | 382944 | 6.25E-05 | 23.94433303 |
| I9-AORTDIS | rs1232604 | 20 | G | A | 0.589704 | -0.256852 | 0.0452715 | 1.40E-08 | 382944 | 8.41E-05 | 32.189473 |
| I9-AORTDIS | rs6024266 | 20 | T | G | 0.288094 | -0.235863 | 0.0515079 | 4.67E-06 | 382944 | 5.48E-05 | 20.96861184 |
| I9-AORTDIS | rs59929908 | 22 | C | T | 0.0727194 | 0.36184 | 0.0790734 | 4.74E-06 | 382944 | 5.47E-05 | 20.93968071 |
| SNP: single nucleotide polymorphism; chr: chromosome; eaf: effect allele frequency; se: standard error of beta. | | | | | | | | | | | |

# Supplementary Figures

| A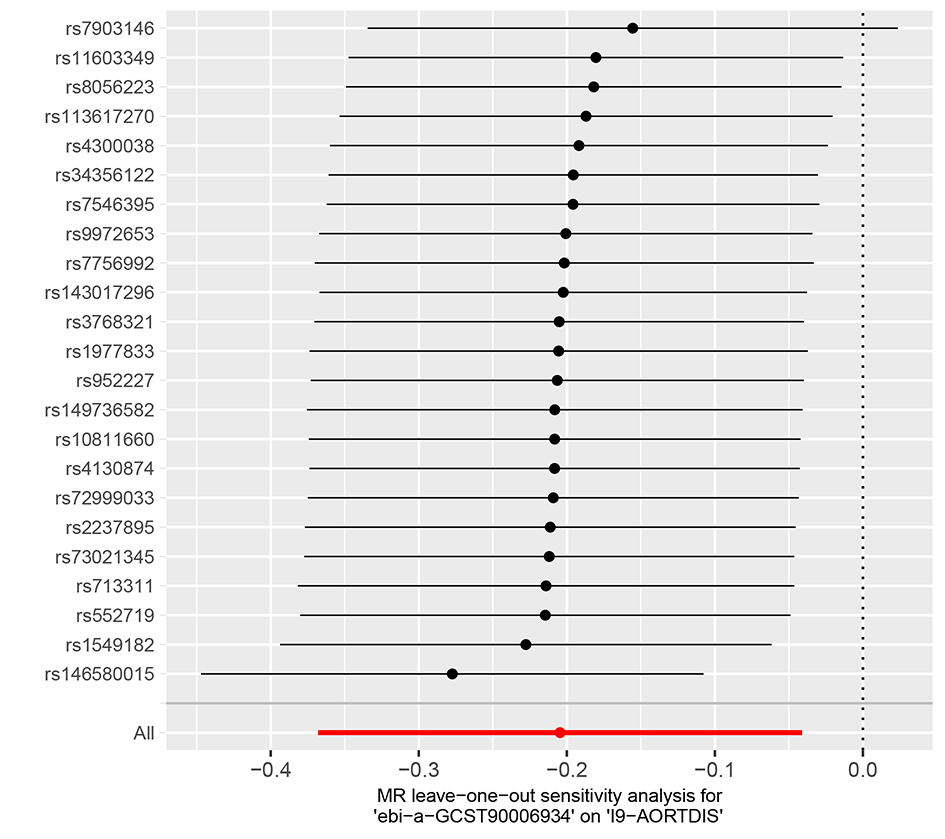 | B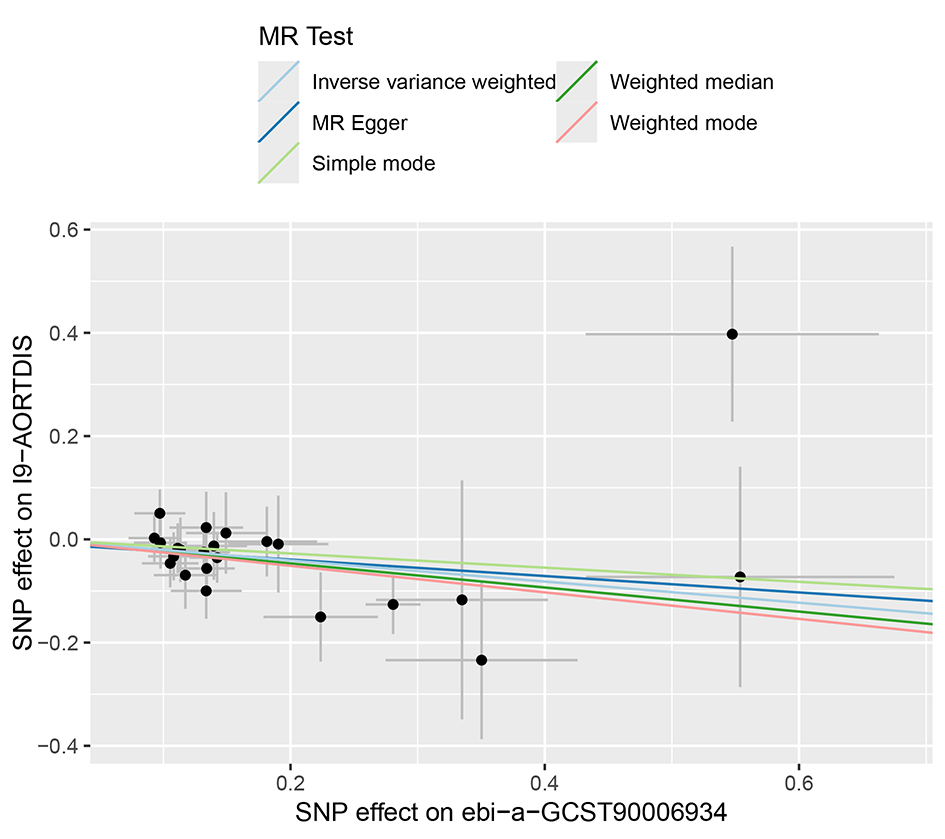 |
| --- | --- |
| C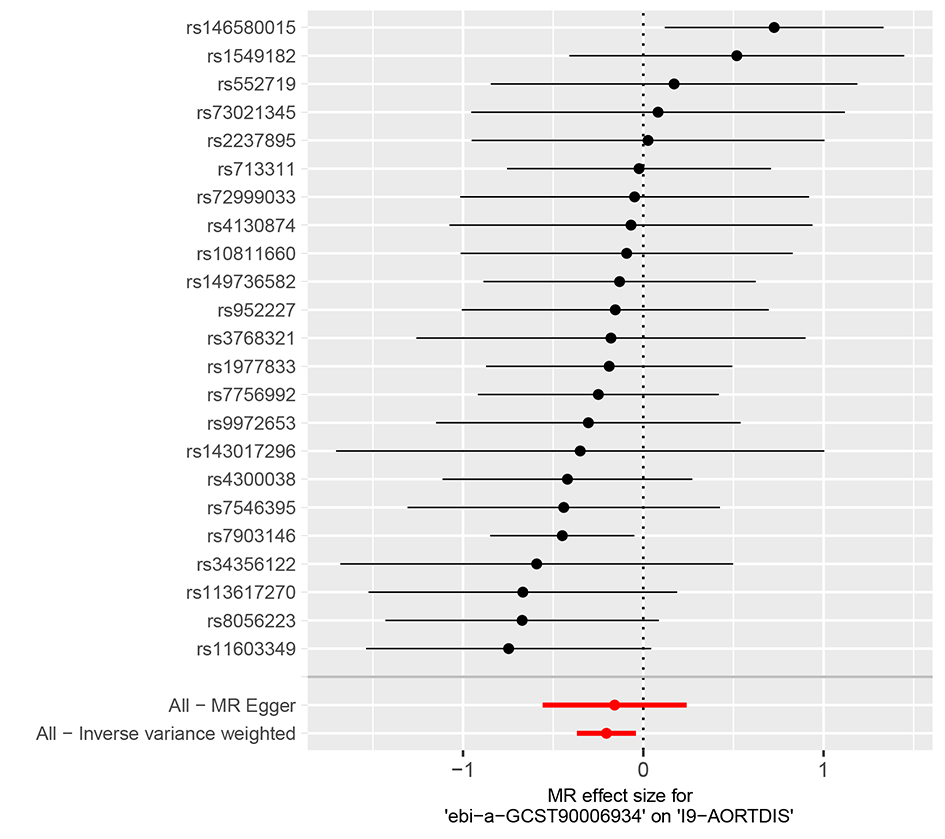 | D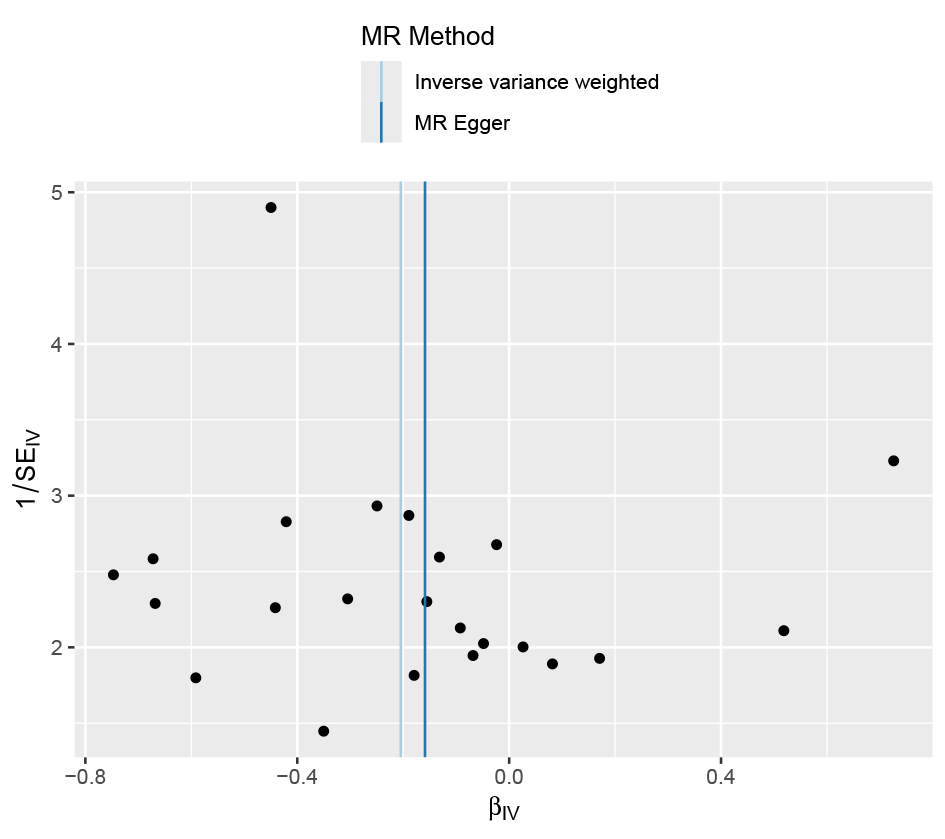 |

**Figure 1.** Forward MR analysis plots of T2DM (ebi-a-GCST90006934) and AD (I9-AORTDIS).

Legend: (A) Leave-one-out plot of T2DM (ebi-a-GCST90006934) and AD (I9-AORTDIS) after removal SNPs one by one. (B) Scatter plot of T2DM (ebi-a-GCST90006934) and AD (I9-AORTDIS). (C) Forest plot of T2DM (ebi-a-GCST90006934) and AD (I9-AORTDIS). (D) Funnel plot of T2DM (ebi-a-GCST90006934) and AD (I9-AORTDIS).

| A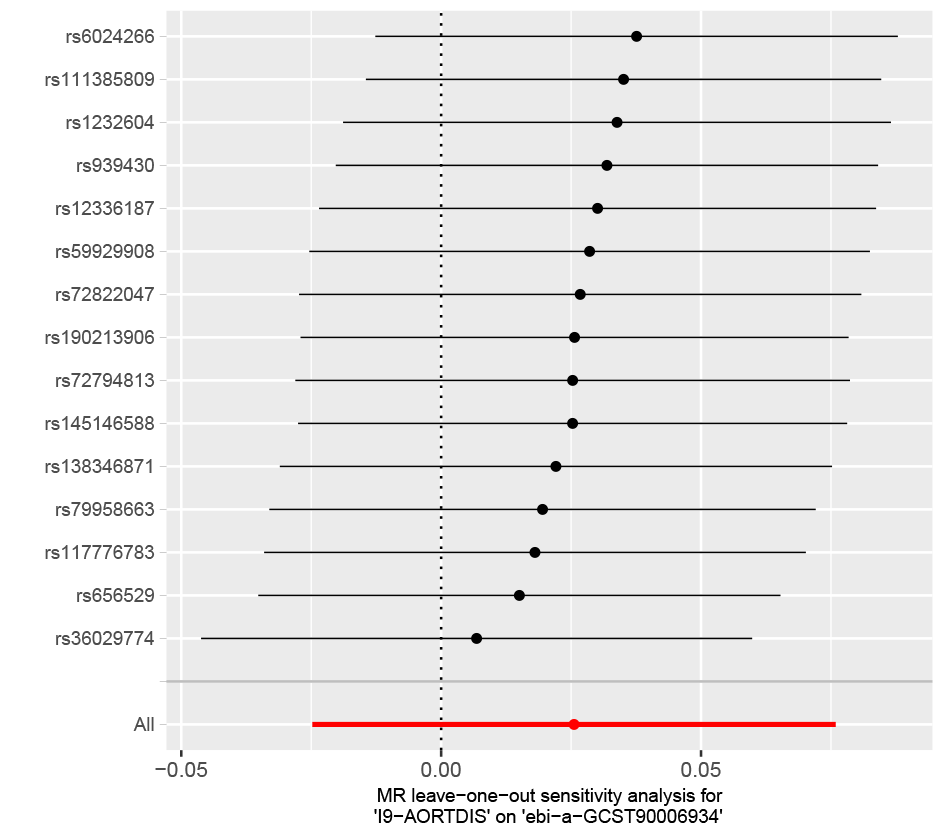 | B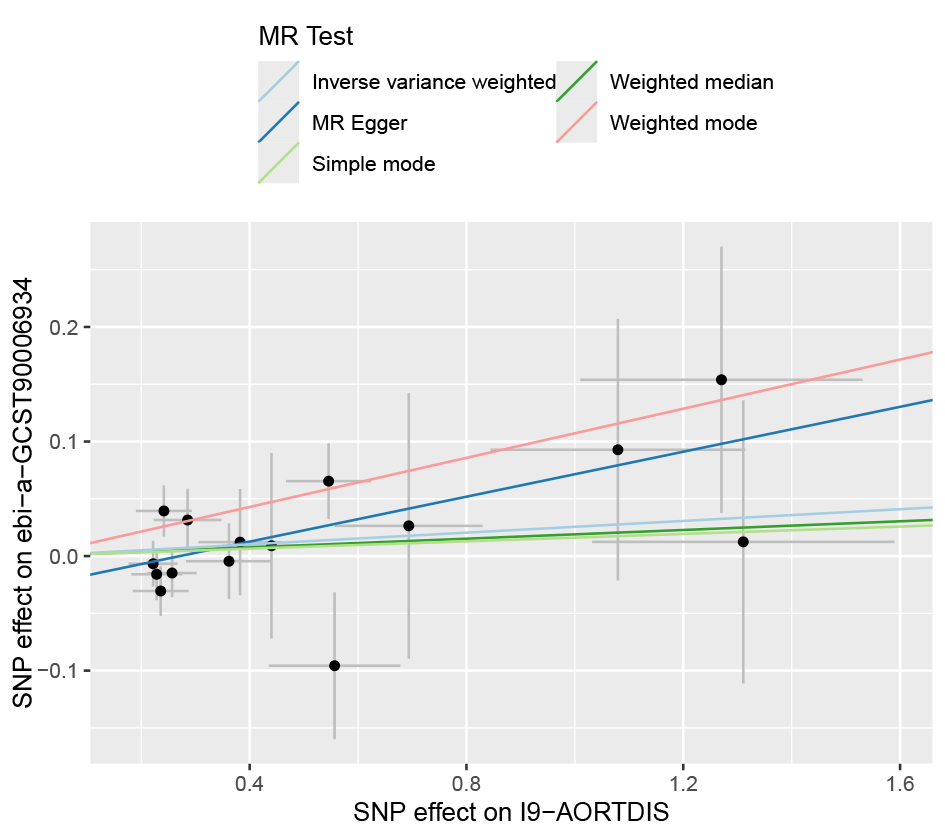 |
| --- | --- |
| C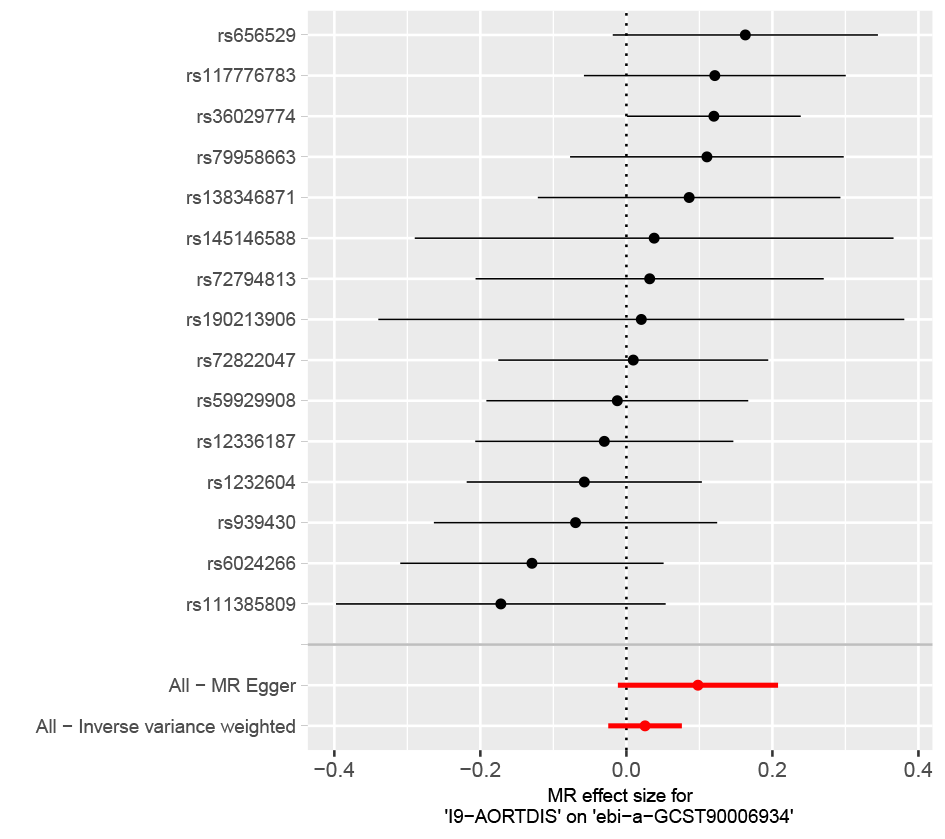 | D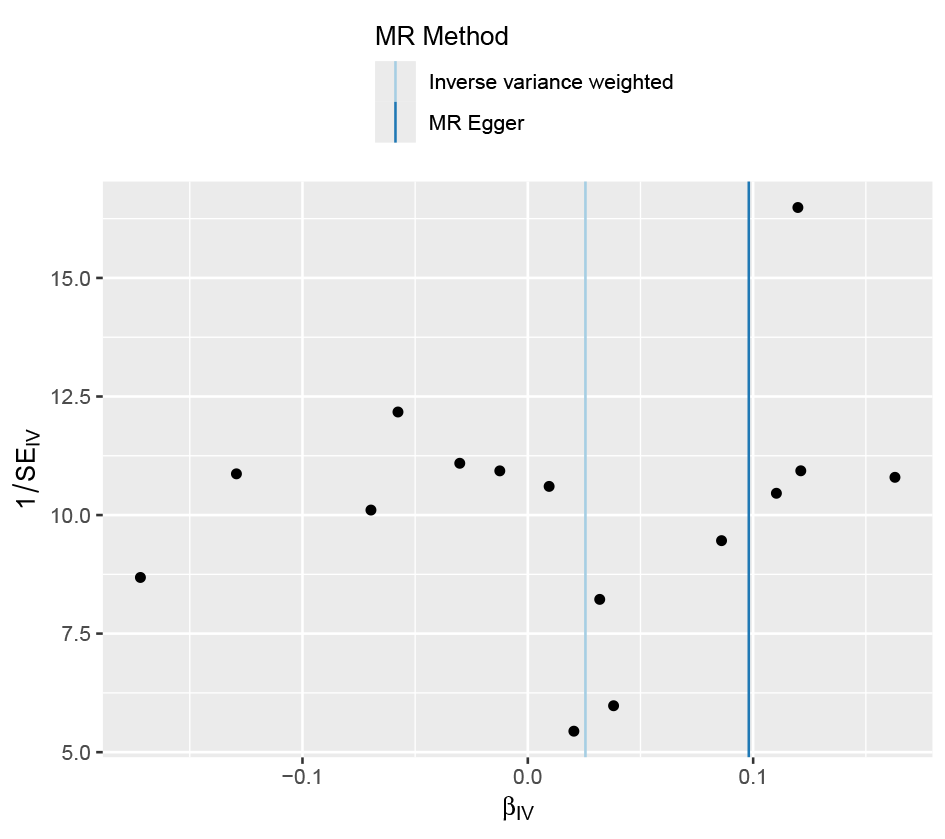 |

**Figure 2.** Reverse MR analysis plots of T2DM (ebi-a-GCST90006934) and AD (I9-AORTDIS).

Legend: (A) Leave-one-out plot of AD (I9-AORTDIS) and T2DM (ebi-a-GCST90006934) after removal SNPs one by one. (B) Scatter plot of AD (I9-AORTDIS) and T2DM (ebi-a-GCST90006934). (C) Forest plot of AD (I9-AORTDIS) and T2DM (ebi-a-GCST90006934). (D) Funnel plot of AD (I9-AORTDIS) and T2DM (ebi-a-GCST90006934).

| A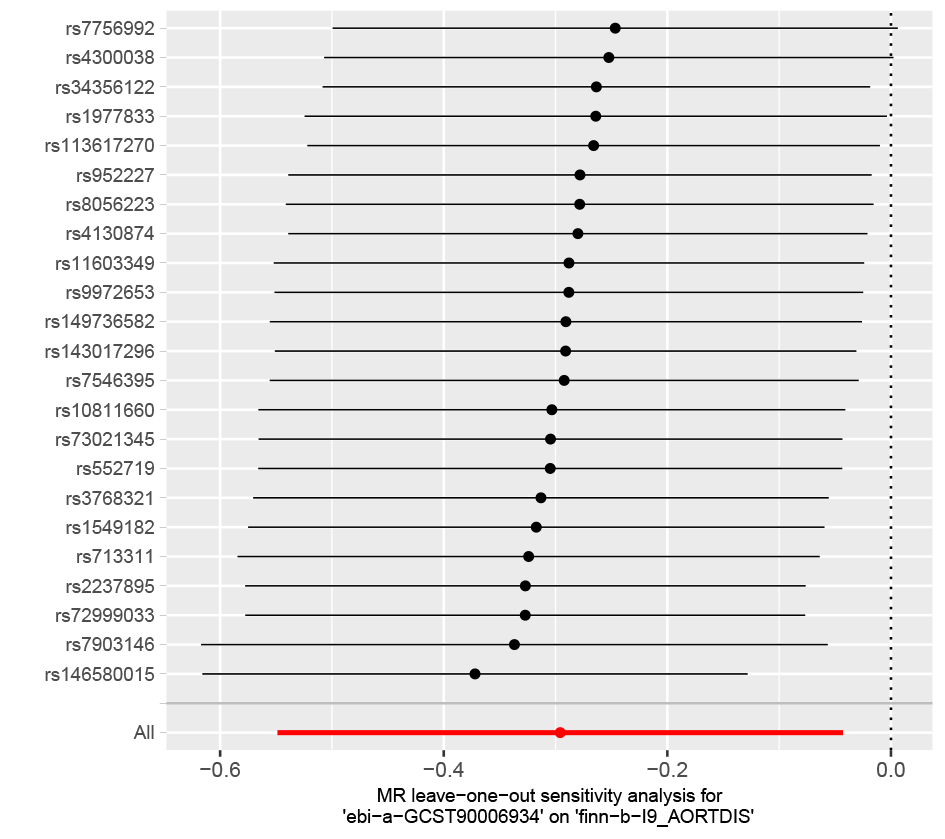 | B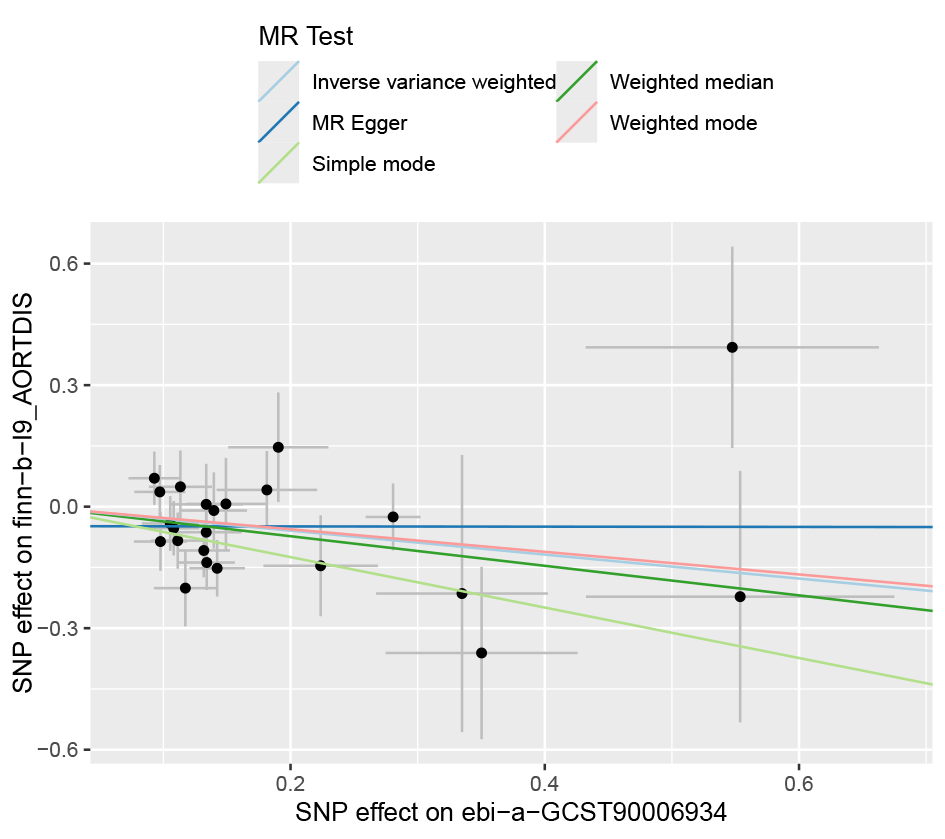 |
| --- | --- |
| C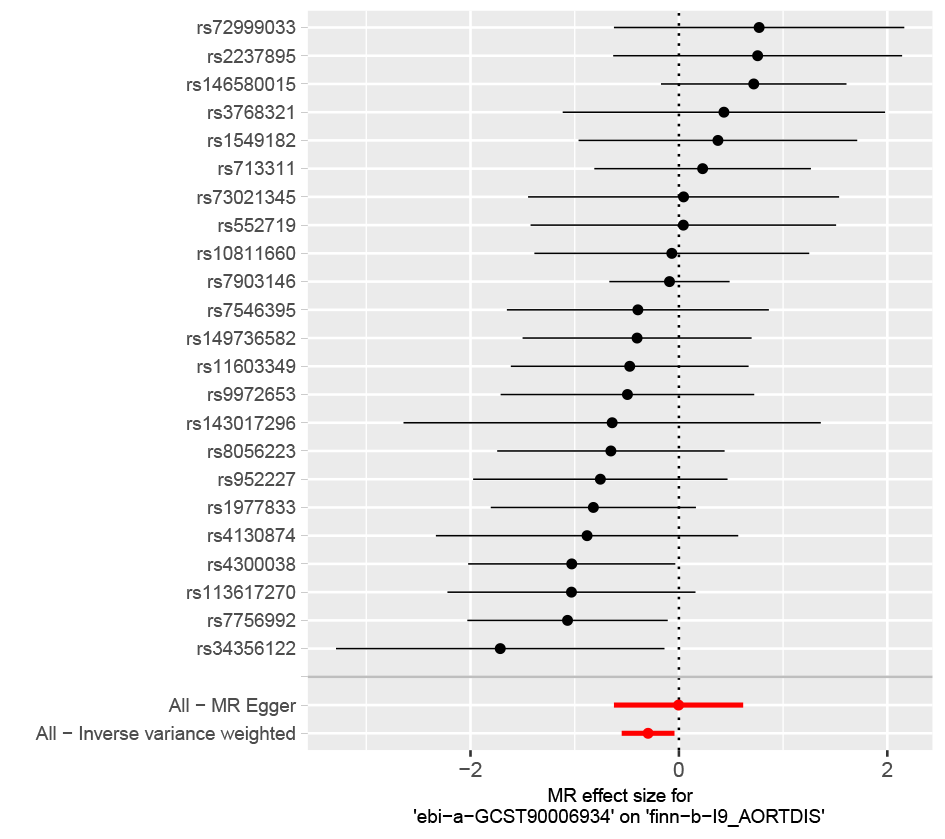 | D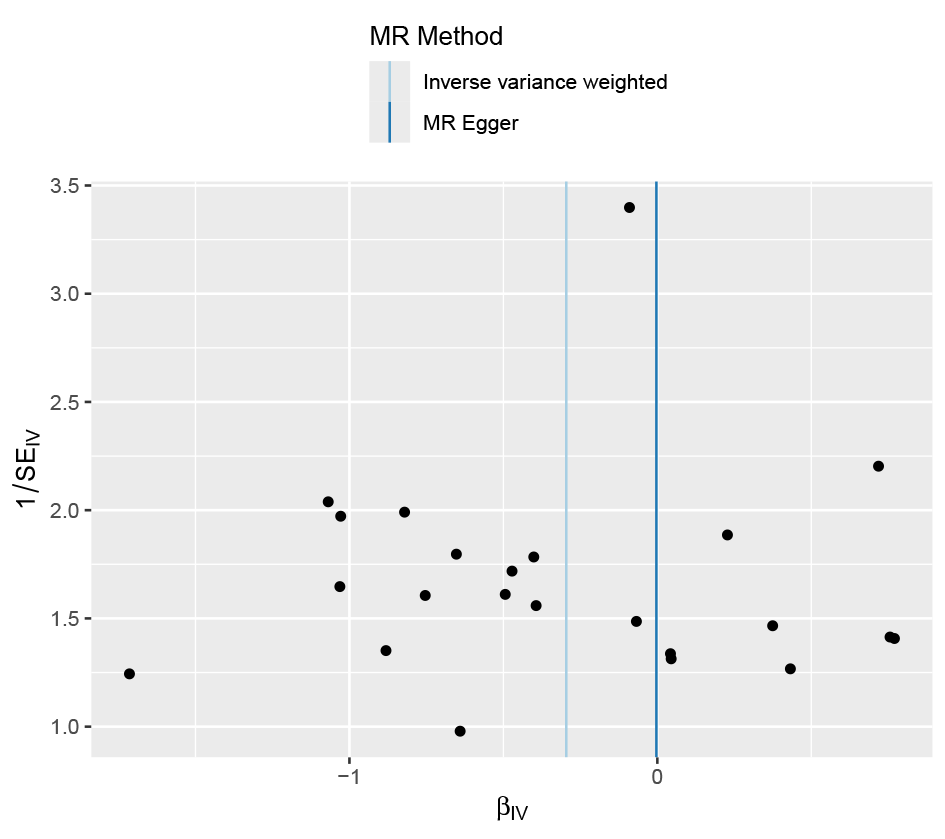 |

**Figure 3.** MR validation analysis plots of T2DM (ebi-a-GCST90006934) and AD (finn-b-I9-AORTDIS).

Legend: (A) Leave-one-out plot of T2DM (ebi-a-GCST90006934) and AD (finn-b-I9-AORTDIS) after removal SNPs one by one. (B) Scatter plot of T2DM (ebi-a-GCST90006934) and AD (finn-b-I9-AORTDIS). (C) Forest plot of T2DM (ebi-a-GCST90006934) and AD (finn-b-I9-AORTDIS). (D) Funnel plot of T2DM (ebi-a-GCST90006934) and AD (finn-b-I9-AORTDIS).
